# Intercellular ultrafast Ca^2+^ wave in vascular smooth muscle cells: numerical and experimental study

**DOI:** 10.1038/srep31271

**Published:** 2016-08-10

**Authors:** J. C. Quijano, F. Raynaud, D. Nguyen, N. Piacentini, J. J. Meister

**Affiliations:** 1 Laboratory of Cell Biophysics, Ecole Polytechnique Fédérale de Lausanne, Lausanne, Switzerland; 2 Facultad de Ciencias Básicas, Politécnico Colombiano JIC, Medellín, Colombia; 3 Computational Systems Oncology, Department of Computational Biology, University of Lausanne, Lausanne, Switzerland

## Abstract

Vascular smooth muscle cells exhibit intercellular Ca^2+^ waves in response to local mechanical or KCl stimulation. Recently, a new type of intercellular Ca^2+^ wave was observed *in vitro* in a linear arrangement of smooth muscle cells. The intercellular wave was denominated ultrafast Ca^2+^ wave and it was suggested to be the result of the interplay between membrane potential and Ca^2+^ dynamics which depended on influx of extracellular Ca^2+^, cell membrane depolarization and its intercel- lular propagation. In the present study we measured experimentally the conduction velocity of the membrane depolarization and performed simulations of the ultrafast Ca^2+^ wave along coupled smooth muscle cells. Numerical results reproduced a wide spectrum of experimental observations, including Ca^2+^ wave velocity, electrotonic membrane depolarization along the network, effects of inhibitors and independence of the Ca^2+^ wave speed on the intracellular stores. The numerical data also provided new physiological insights suggesting ranges of crucial model parameters that may be altered experimentally and that could significantly affect wave kinetics allowing the modulation of the wave characteristics experimentally. Numerical and experimental results supported the hypothesis that the propagation of membrane depolarization acts as an intercellular messenger mediating intercellular ultrafast Ca^2+^ waves in smooth muscle cells.

Communication between vascular smooth muscle cells (SMCs) plays an important role in coordinating vascular function and compromised intercellular signaling may underlie pathological conditions. Continuous electrical and ionic movements take place between coupled cells which affect resting states and enable conduction of signals. Electrical current, inositol 1,4,5-trisphosphate (IP_3_) and Ca^2+^ are considered as important mediators of vascular communication. Nevertheless, Ca^2+^ and IP_3_ fluxes through gap junctions are small and thus, their passive diffusion should have a limited effect on Ca^2+^ mobilization at distant sites^1^. One way of cellular communication is by intercellular Ca^2+^ waves, the propagation of an increase in intracellular Ca^2+^ concentration. Such intercellular Ca^2+^ waves have been induced *in vitro* by mechanical, electrical or chemical stimuli^2^,^3^,^4^ and classified according to the mechanism involved and the velocity amplitude, denominating the ultrafast Ca^2+^ wave as an electrically propagated wave^5^,^6^. Novel insights have been gained from mathematical models which connect clusters of SMCs^7^,^8^,^9^,^10^,^11^. In particular, in ref. 11 the authors confirmed the hypothesis that intercellular Ca^2+^ waves observed in arterial SMCs^12^ resulted from electrical coupling. Assuming gap junctional communication by means of electrical coupling, IP_3_ diffusion, and Ca^2+^ diffusion these models reproduced experimental observations like asynchronous Ca^2+^ flashings, recruitment of cells and vasomotion in absence of endothelium^13^,^14^,^15^,^16^,^17^. In the present study, we adapted the model presented in ref. 11 to elucidate the mechanisms underlying the ultrafast Ca^2+^ wave and to investigate the particular conditions for intercellular ultrafast Ca^2+^ wave to occur as well as the properties of the membrane depolarization. Our study showed the direct interplay between the Ca^2+^ wave and the spreading of the membrane depolarization. We tested, discussed and demonstrated that an intercellular ultrafast Ca^2+^ wave is driven by the propagation of cell membrane depolarization and its speed is not dependent on the intracellular Ca^2+^ stores. Simulations predicted novel results and opened the field for further experimental studies to investigate the effect of electrical coupling and whole-cell conductance on Ca^2+^ wave velocity and on the propagation speed of membrane depolarization.

## Results

### Propagation of the induced intercellular ultrafast Ca^2+^ wave and induced membrane depolarization

**Figure 1:**
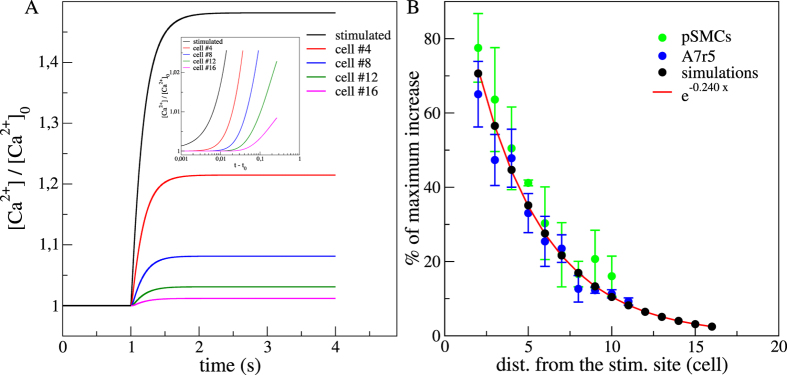
(**A**) Numerical results for the time courses of Ca^2+^ responses in the simulated control case. Stimulation occurs at 1 s. Stimulated cell (black), 4th cell (red), 8th cell (blue), 12th (green) and 16th cell (pink). [*Ca*^2+^]_0_ is the steady state cytosolic [Ca^2+^] before stimulation. Inset presents the short time dynamics after stimulation (*t*_0_) in lin-log scale. (**B**) Percentages of maximum *F*/*F*_0_ (n_*pSMCs*_ = 16; n_*A*7*r*5_ = 19) and [Ca^2+^]/[Ca^2+^]_0_ increases, assuming 100% as the maximum level reached by the stimulated cell. Red line fits the exponential decrease with distance from the stimulated site and cell unit is estimated by taking a cell of 150 *μ*m. (*n*_*pSMCs*_ = 22; *n*_*A*7*r*5_ = 82).

For the set of parameters corresponding to the numerical control case (see Methods), the time evolution of the [Ca^2+^], normalized by the steady state concentration before activation ([Ca^2+^]_0_), is depicted in Fig. 1A. Before the stimulation (t < 1 s), all cells were at the same resting state. After the stimulation, we observed a global Ca^2+^ increase and each cell reached a new steady state with an asymptotic [Ca^2+^] that decreased exponentially with the distance from the stimulated site. We measured a typical scale of 4,16 cells (*R*^2^ = 0,99) (Fig. 1B). Experimentally, we found a similar exponential relationship between the percentage of maximum increase and the distance from the stimulation site with a typical scale of 4,4 cells (
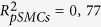
; 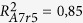). A closer look to the short time dynamics just after the stimulation showed a lag in the Ca^2+^ increase as an indication of an intercellular Ca^2+^ wave. To estimate the onset times and calculate the wave velocity, we measured for each cell, the time required to reach different thresholds from 1% to 15% of its maximum value of [Ca^2+^]. For each threshold, we determined the velocity from the difference in the onset times between one particular cell to the following cell and then averaged over all the cells and for all threshold values. We obtained a wave velocity of ~333(±40) cell.s^−1^.

**Figure 2:**
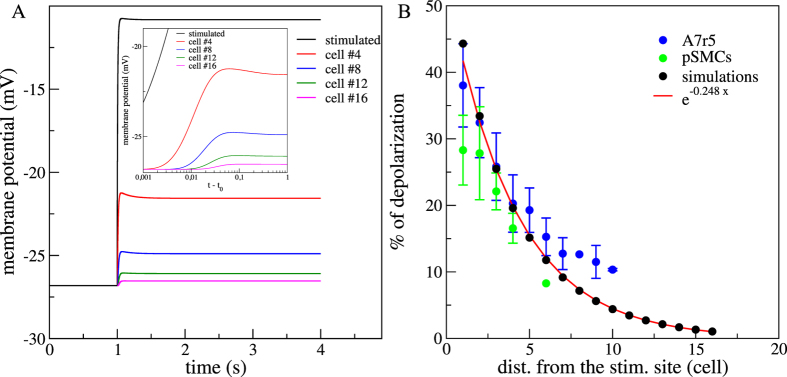
(**A**) Numerical results for the time courses of the membrane potential in the simulated control case. Stimulation occurs at *t* = 1 *s*. Stimulated cell (black), 4th cell (red), 8th cell (blue), 12th (green) and 16th cell (pink). Inset presents the short time dynamics after stimulation (*t*_0_) in lin-log scale. (**B**) Percentages of membrane depolarization (n_*pSMCs*_ = 16; n_*A*7*r*5_ = 19). The percentages of membrane depolarization were calculated using the maximum depolarization value of each cell and comparing it with the steady state membrane potential before stimulation. Red line fits the exponential decrease with distance from the stimulated site and cell unit is estimated by taking a cell of 150 *μ*m. (*n*_*pSMCs*_ = 22; *n*_*A*7*r*5_ = 82).

Similarly as the *in vitro* experiments reported in ref. 18, numerical results showed that membrane potential increased after stimulation. Maximum of the depolarization was greater for cells close to the stimulated one (Fig. 2A). We calculated the percentage of membrane depolarization using the maximum depolarization value of each cell with respect to the steady state membrane potential before the stimulation. Figure 2B shows that the percentage of membrane depolarization followed an electrotonic behaviour with exponential decrease. We obtained a characteristic lenght scale of 4,03 cells (*R*^2^ = 0,98) in good agreement with previously reported experimental data (assuming a cell length of 100–150 *μ*m) for vascular SMCs 4,46–6,13 cells^18^.

### Role of gap junctions channels and VOCCs in the simulated array of SMCs

Recent studies showed that incubation of the SMCs *in vitro*^3^,^18^ or arterial strips^12^ with gap junction uncoupler palmitoleic acid (PA) or with VOCCs inhibitor nifedipine totally abolished the Ca^2+^ waves. In relation to these experimental results, we simulated similar conditions by modification of the parameters governing the dynamics of these channels (g and G_Ca_, see Methods). We first simulated a total inhibition of the gap junctions by setting the electrical coupling coefficient *g* to 0.

**Figure 3:**
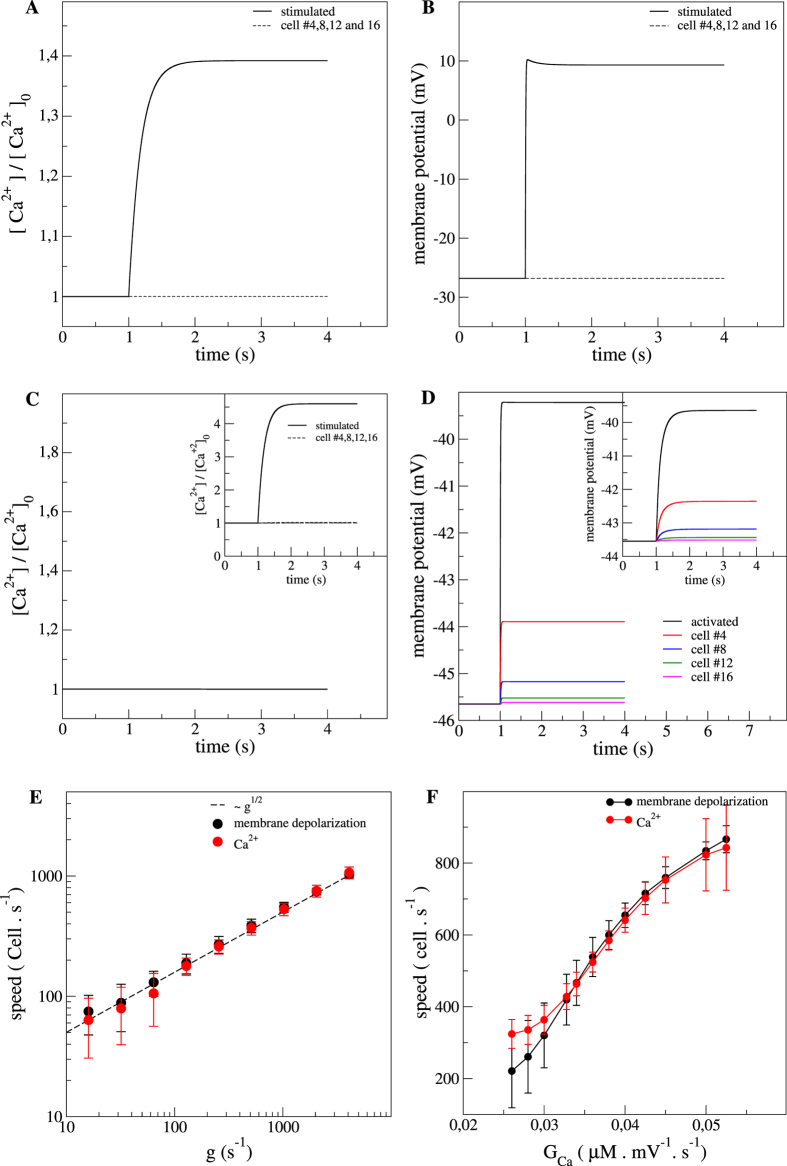
Role of gap junctions and VOCCs on the Ca^2+^ dynamics and membrane potential. (**A**) Time evolution of the Ca^2+^ responses and (**B**) membrane potential variations with inactivated gap junctions. The black dashed line is the response of the other cells but the stimulated one. Gap junctions inhibition was simulated by setting the electrical coupling coefficient (*g*) to 0 s^−1^. (**C**) Time evolution of the Ca^2+^ response and (**D**) membrane potential variations with inactivated VOCCs. VOCCs inhibition was simulated by setting the whole-cell conductance to 0 mV^−1^.s^−1^. (**E**) Theoretical Ca^2+^ wave velocity (red) and propagation speed of membrane depolarization (black) as a function of *g*. The dashed line represents the scaling g^1/2^. (**F**) Ca^2+^ wave velocity (red) and propagation speed of membrane depolarization (black) as a function of G_Ca_. Inset in (**C**) is the time evolution of the Ca^2+^ response and the inset in (**D**) is membrane potential variations with an impose Ca^2+^ transient in the first cell.

As in *in vitro* conditions^3^,^18^, we observed a total suppression of the Ca^2+^ and the membrane potential signals under gap junctions inactivation. Only the stimulated cell showed a Ca^2+^ increase and a membrane depolarization; responses of the other cells of the network were insignificant (dashed line in Fig. 3A,B). We extended the analysis for a wide range of electrical coupling constants (Fig. 3E) and observed that both the speed of the Ca^2+^ wave and the propagation speed of membrane depolarization increased like the square root of the coupling, in a similar way as in a system of coupled oscillators. Within this range of electrical coupling, we obtained Ca^2+^ wave speeds from 46 cell.s^−1^ to 866 cell.s^−1^ and membrane depolarization conduction speeds from 60 cell.s^−1^ to 833 cell.s^−1^.

We simulated VOCCs blockage by imposing a null whole-cell conductance G_Ca_ = 0. This condition inhibited any Ca^2+^ entry through VOCCs anywhere along the SMCs network, including the stimulated cell, resulting in the abolition of the Ca^2+^ wave (Fig. 3C). Importantly, there was still a membrane depolarization spreading along the network even in zero VOCCs conductance conditions (Fig. 3D). In the simulations, none of the cells had a [Ca^2+^] response for G_Ca_ = 0, whereas in the experiments with nifedipine the stimulated cell had (in response to the activation of other channels such as stretch activated channels). To ensure that in the simulations the abolition of the ultrafast [Ca^2+^] wave was not a trivial consequence of the absence of [Ca^2+^] increase of the stimulated cell, we designed a numerical experiment in which we imposed a [Ca^2+^] profile to the first cell (insets in Fig. 3C,D). In this situation, the other cells did not respond to the imposed [Ca^2+^] increase whereas a spreading of membrane depolarization still occurred in the system (inset in Fig. 3D). These numerical results were an important milestone of the current study and were crucial to confirm the hypothesis that [Ca^2+^] increase over the network of SMCs was fundamentally due to the Ca^2+^ entry through the opening of VOCCs in response to a propagation of membrane depolarization along the SMCs network. Beside the condition of null whole-cell conductance, we also investigated the effect of G_Ca_ on the speeds of the [Ca^2+^] wave and membrane depolarization propagation and observed a non trivial increase with G_Ca_.

### Role of the intracellular Ca^2+^ stores (IP_3_ and Ryanodine receptors) in the simulated array of SMCs

**Figure 4:**
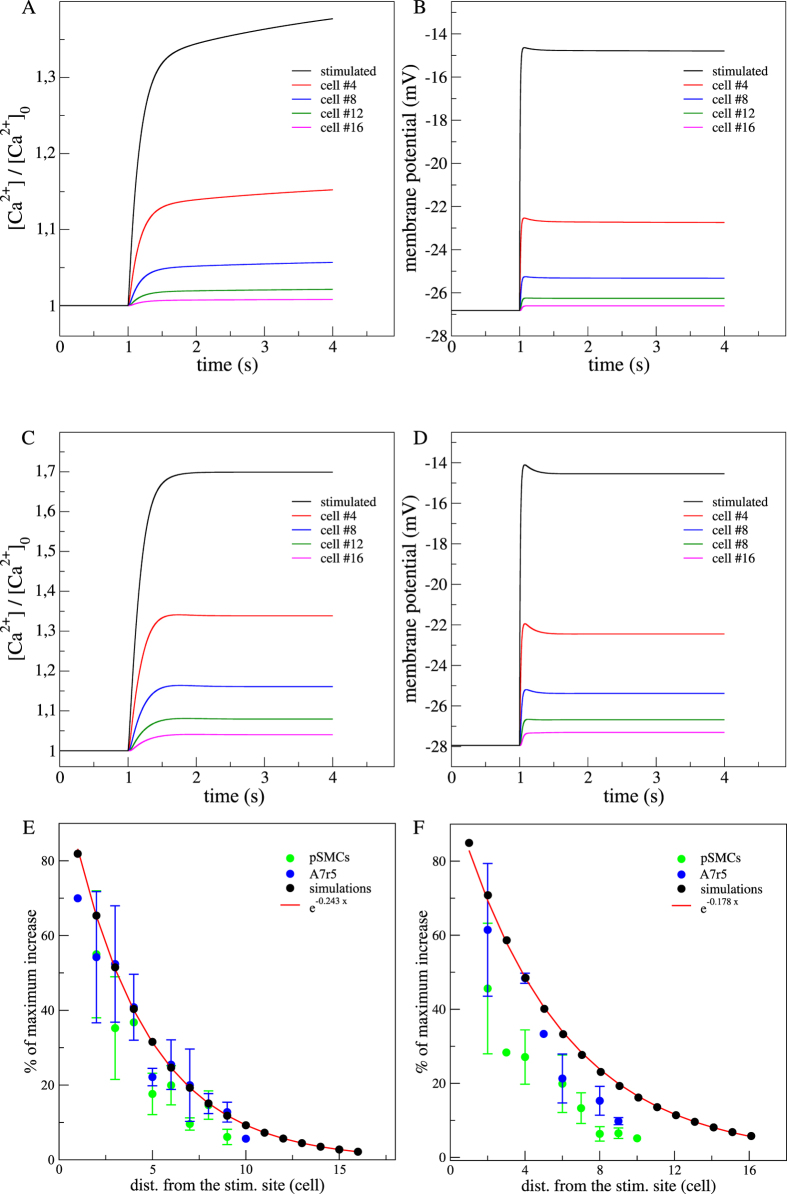
Effects of RyRs or IP_3_Rs inhibition in the Ca^2+^ dynamics and membrane potential in a simulated array of SMCs. (**A**) Time evolution of the Ca^2+^ response and (**B**) membrane potential variations along the network SMCs with inactive RyRs. RyRs inhibition was simulated by diminishing the CICR rate constant (*C*) to 0 *μ*m.s^−1^. (**C**) Time evolution of the Ca^2+^ response and (**D**) membrane potential variations with inactive IP_3_Rs. IP_3_Rs inhibition was simulated by diminishing the maximal rate of activation-dependent Ca^2+^ influx (*F*) to 0 *μ*m.s^−1^. Stimulated cell (black), 4th cell (red), 8th cell (blue), 12th cell (green) and 16th cell (pink). (**E**) Percentages of maximum *F*/*F*_0_ and [Ca^2+^]/[Ca2+]_0_ increases with inactive RyRs (*n_pSMCs_* = 4, *n_A7r5_* = 4) and (**F**) with inactive IP_3_Rs (*n_pSMCs_* = 5, *n_A7r5_* = 5). Red line fits the exponential decrease with distance from the stimulated site.

In previous experimental studies^3^,^18^, results obtained using ryanodine showed that the magnitude of the Ca^2+^ wave was unaffected in linear arrangements of cultured vascular SMCs (see Supplementary Fig. S1E). This suggested that the Ca^2+^ induced Ca^2+^ release mechanism (CICR), through the RyRs, did not substantially contribute to the mechanism of ultrafast Ca^2+^ wave propagation, nor to the rate and amplitude of the [Ca^2+^] increase. Nevertheless, in the case of a minimal CICR, this event would be subsequent to the onset of the [Ca^2+^] increase and then would not affect drastically the ultrafast Ca^2+^ wave velocity. We simulated experiments inhibiting each of the two intracellular Ca^2+^ sources by modifying *F* for the Ca^2+^ release from IP_3_ sensitive stores and *C* for the CICR mechanism (Equations 9 and 12). Comparison between simulations under inactive Ryrs (Fig. 4A,B) and numerical control case (Figs 1A and 2B) did not show any significant changes in the Ca^2+^ and membrane depolarization responses.

The other source of Ca^2+^ release from intracellular Ca^2+^ stores that could be involved was the IP_3_Rs path. We investigated whether IP_3_Rs did play a role in the generation of the ultrafast Ca^2+^ wave by incubating the two types of vascular SMCs with 30 *μ*M 2-APB for 10 minutes. The inhibition of IP_3_ receptors with 2-APB caused a significant decrease in the average fluorescence amplitude in the stimulated cell (43,4 ± 9.2%, *n*_*pSMC*_ = 5; 50 ± 12.5%, *n*_*A*7*r*5_ = 5), but did not suppress the propagation (see Supplementary Fig. S1F) nor the wave magnitude of the ultrafast Ca^2+^ wave (91,3 ± 22cell.s^−1^, *n*_*pSMCs*_ = 5; 94 ± 22cell.s^−1^, *n*_*A*7*r*5_ = 5). This new experimental result supported the postulated hypothesis that the Ca^2+^ release through IP_3_Rs was not involved in the ultrafast Ca^2+^ wave mechanism. Altogether the experimental and theoretical results obtained by blocking the intracellular stores, demonstrated the hypothesis that the CICR did not contribute significantly to the mechanism of the ultrafast Ca^2+^ wave propagation nor did it to the fast Ca^2+^ increase process along the SMCs network.

### Conduction velocity of the mechanically-induced membrane depolarization

**Figure 5:**
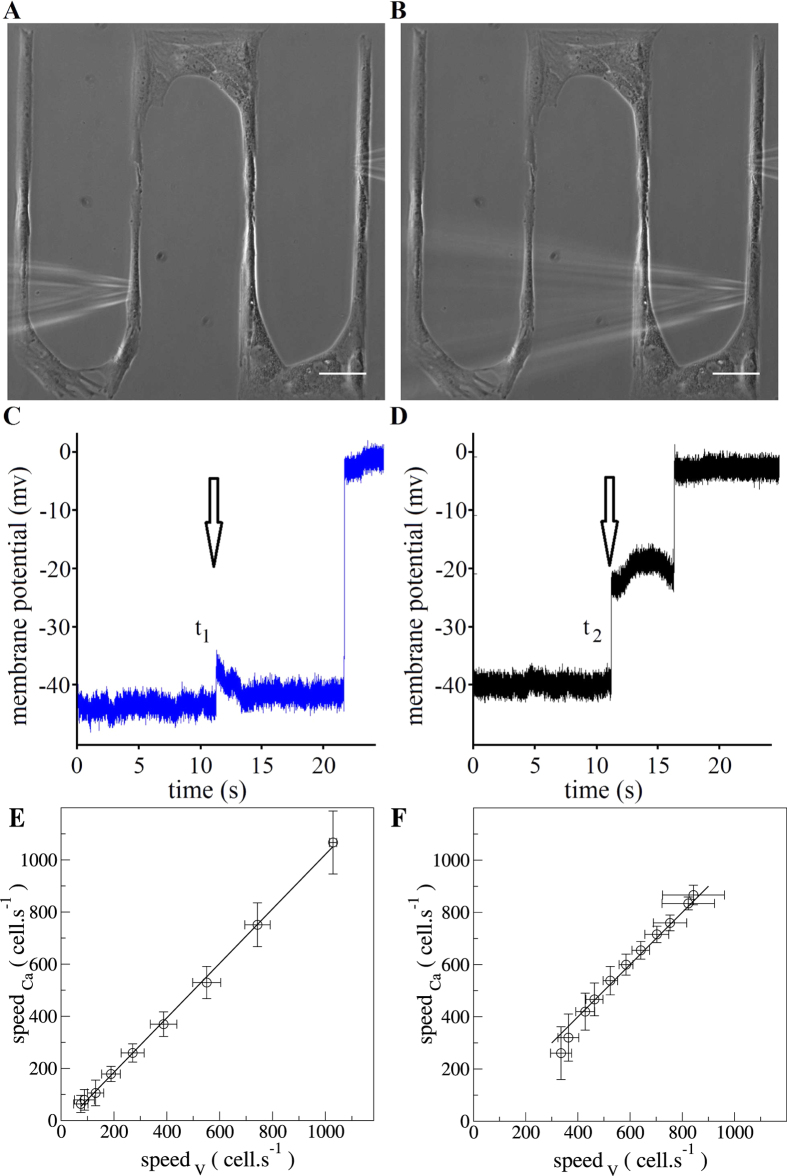
Representative conduction velocity approach in a linear arrangement of SMCs. Phase contrast images showing the spatial arrangement of the microelectrode (left) and the micropipette (right) in the (**A**) first and in the (**B**) second mechanical stimulation attempt. Scale bar: 50 *μ*m. Typical membrane potential recordings in a cell during mechanical stimulation in another cell for the (**C**) first and for the (**D**) second attempt. The conduction velocity was calculated by dividing the separation distance between the two impaled cells and the time delayed between the two membrane potential registrations (*t*1–*t*2). The arrows in the (**C**,**D**) panels indicate the moment when the mechanical stimulation was launched. Theoretical relation between the Ca^2+^ wave velocity and the propagation speed of membrane depolarization varying *g* (**E**) or G_Ca_ (**F**).

For the first time, we estimated the spreading velocity of the membrane depolarization for two types of vascular SMCs using intracellular membrane potential recording with the microelectrodes technique combined with precise patterning technique. Experimentally, two identical mechanical stimulations in the same cell and two consecutive successful impalements in two different cells in the same network allowed to calculate the conduction velocity of the membrane depolarization along the network (Fig. 5A–D). The experimental conduction velocity obtained in linear networks of cultured vascular SMCs was 98 ± 27,3 cell.s^−1^ (*n* = 3) for pSMCs and 94,1 ± 24 cell.s^−1^ (*n* = 4) for A7r5 and were not statistically different (*P* > 0,05). Numerically, we also measured the conduction velocity by extracting the onset times at which the depolarization reached 50% of its maximal level. For the values of the coupling constant *g* and whole-cell conductance G_Ca_ under study, we confirmed the linear relationship between the speed of the ultrafast Ca^2+^ wave and the conduction speed of the membrane depolarization (Fig. 5E,F). This suggests that in principle under different physiological conditions, it is possible to infer one velocity from the knowledge of the other.

## Discussion

This study focused on the mechanism of propagation of intercellular ultrafast Ca^2+^ waves in vascular smooth muscle cells resulting a from single cell membrane depolarization.

Our findings indicated that the propagation of membrane depolarization mediated by the electrical coupling between cells was at the origin of the observed ultrafast Ca^2+^ waves whereas intracellular and intercellular diffusion mechanisms did not play a significant role. In the numerical control case, the value of the velocity was found within the correct order of magnitude but slightly overestimated compared to experimental results. Different factors may explain the difference between the numerical and experimental wave velocities, like model parameters (that correspond to *in vivo* conditions whereas experiments were conducted *in vitro*) or experimental conditions (the temperature might affect biological rate processes up to two times for a change of 10 °C^19^). Nevertheless, the exact experimental values of the Ca^2+^ and depolarization velocities were found for smaller electrical coupling (or whole cell conductance) in a region of the parameter space that may correspond to a situation closer to *in vitro* conditions.

Membrane depolarization resulted from outward chloride currents through Ca^2+^ -activated chloride channels (Equation 14) and propagated along the SMCs network in an electrotonic manner with an exponential decay of 4 cells ranging within the experimental values for the two types of SMCs^18^. Most experimental evidences suggested that conducted responses depended primarily on passive electrotonic spread^1^ and this feature was also found to be essential in intact artery^11^. The flux of Ca^2+^ through VOCCs depended on the voltage and decayed exponentially along the network. Consequently, a similar decay for the maximal Ca^2+^ increase was expected. Numerically and experimentally we found that the space constants for the percentage of depolarization and the level of maximum Ca^2+^ increase were similar, confirming these two processes to be linked. This behaviour was observed after chemical stimulation in endothelial cells^20^, local mechanical stimulation in epithelial^21^ and in primary SMCs^3^.

Our numerical model allowed to explore the behavior of ultrafast Ca^2+^ waves beyond standard experimental conditions. Setting the electrical coupling coefficient to zero completely inhibited the wave as in experiments with a gap junction inhibitor^3^,^18^. This abolition has also been shown in simulated regenerative Ca^2+^ waves^11^ and experimentally in arterial strips^12^. We predicted the Ca^2+^ wave speed to increase as the square root of the electrical coupling parameter. This result could be further tested experimentally by increasing the electrical coupling with drugs used for acute opening of gap junctions^22^ or by imposing growth arrested conditions in cell preparations (that had been reported to enhance gap junctions permeability^23^). Therefore, we provided potential insights for further research in the field of gap junction proteomics, suggesting a possible modulation of Ca^2+^ wave velocity probably due to alterations of the connexins expression in the gap junctions. Setting VOCCs conductance to zero completely inhibited the Ca^2+^ wave; only the electrotonic propagation of membrane potential to neighboring cells was preserved. This confirmed that VOCCs were the main source of Ca^2+^ influx and the principal actor for the [Ca^2+^] increase during mechanical or KCl stimulation. Simulations predicted an increase of the velocity of the ultrafast Ca^2+^ in response to an increase of the VOCCs conductance. Our result was qualitatively in agreement with simulated regeneative Ca^2+^ waves, where a linear increase of the wave velocity had been observed for moderate values of G_Ca_^11^. Interestingly, we found a nonlinear increase for higher values of VOCCs conductance. This numerical result was important because it is extremely challenging to modify experimentally the whole-cell conductance of VOCCs. Assuming that the effect of a VOCCs activator (i.e. Bay K8644) would be an increase in whole-cell conductance of VOCCs, we could correlate experiments in the presence of this drug with simulations at an elevated VOCCs conductance. Experimentally, it has been found that the Ca^2+^ wave was enhanced during Bay K8644 treatment in arterial strips^12^. In cultured rat mesenteric SMCs the incubation with Bay K8644 resulted in a longer [Ca^2+^] increase but not in a higher Ca^2+^ wave velocity^3^. It may be not suitable experimentally to obtain higher VOCCs conductance values only by the addition of elevated concentrations of Bay K8644. This result could be due to the fact that Bay K8644 may not increase drastically the VOCCs whole-cell conductance and highlighted the difficulty to predict experimentally the consequences of a gradual change of the VOCCs conductance. Therefore, this new numerical result opens the possibility to search and study new mechanisms in which one could not only enhance the Ca^2+^ wave, but also manipulate and tune the velocity of the ultrafast wave experimentally.

We provided further experimental evidences and confirmed numerically that the ultrafast Ca^2+^ wave was unaffected, at least in its onset, by the inhibition of each of the two main sources of intracellular Ca^2+^^18^. Simulations showed that setting the CICR rate constant to zero did not have a major effect in propagation of the Ca^2+^ wave. In this way, the *in vitro* system differed from the *ex vivo* case, where the CICR was essential for Ca^2+^ flashes and zero CICR rate constant abolishes the Ca^2+^ wave^11^. The other physiologically possible intracellular Ca^2+^ source is the IP_3_Rs. In agreement with experiments, the blockade of the IP_3_ mediated Ca^2+^ release did not affect the Ca^2+^ nor the membrane potential responses. In our systemm the stimulation was not based on background agonist. Consequently the IP_3_ levels remained constant and then no Ca^2+^ release occurred through this path, indicating that intracellular Ca^2+^ stores were not involved in the mechanism of the ultrafast Ca^2+^ wave. Furthermore, according to previous Ca^2+^ waves description and classification, most of the Ca^2+^ waves with a speed >10 mm.s^−1^ were not governed by intracellular Ca^2+^ processes^5^,^6^.

In a tissue made of electrically unexcitable cells, a change in the membrane potential could electrotonically propagate intercellularly with an exponential decrease in amplitude over distance. This coupling can only extend a few millimeters but can be very rapid (a few milliseconds)^24^. We estimated a conduction velocity that was in the same order of magnitude than previous values obtained for different experimental models such as guinea pig ureter (153 cell.s^−1^)^25^ and small intestine (513–586 cell.s^−1^)^26^. As suggested, the propagation of the membrane depolarization can be sufficient to coordinate smooth muscle cell relaxation within and among branches of vascular resistance networks^27^. Although the electrical signal that propagated in the linear arrangements of SMCs is not an action potential, the membrane potential depolarization must pass through the same gap junctions as for action potentials like for spontaneously electrical waves (known as slow waves) in the Gastric Antrum^28^,^29^,^30^,^31^ and in airway SMCs^32^. This indicated that the speed of propagation of these two phenomena should be directly correlated and the ultrafast Ca^2+^ wave was most probably a consequence of the membrane depolarization spreading. Nonetheless, if a delay between the spreading of the depolarization and the initial entry of the Ca^2+^ through VOCCs was expected, it would be conserved for the whole network without affecting the velocities. Comparison between the velocities of the ultrafast Ca^2+^ wave and the membrane depolarization propagation revealed no statistical difference for both types of vascular SMCs. Altogether these results suggest that it is likely that the velocity of the ultrafast Ca^2+^ wave was determined by the propagation speed of the membrane depolarization followed by Ca^2+^ entry through VOCCs. Similarly to gastrointestinal electrical slow waves propagation^31^, the wave range of the ultrafast Ca^2+^ wave in cultured vascular SMCs was not limited by the diffusion of Ca^2+^ in relation to conduction by CICR. On the contrary, it was mediated by current spread that had more spatial influence compared to the diffusion of second messengers of Ca^2+^ or IP_3_. Moreover, the model predicted that the direct relation between those two phenomena held for a wide range of electrical coupling coefficient and whole cell conductance for VOCCs. This fact, may endorse new experimental approaches to explore the physiological consequences in the vasculature.

## Conclusion

In this work we provided an accurate framework to investigate the behavior of ultrafast Ca^2+^ wave under various physiological conditions, including extreme physiological conditions where wave properties could only be extrapolated from experiments. Conceptually, the present approach represented a significant advance by combining new theoretical and new experimental data for both Ca^2+^ and membrane potential dynamics. Numerically, we found that the ultrafast Ca^2+^ was not regenerated, it had a velocity and a spatial range in agreement with experiments. We validated the electrotonic behavior of the membrane depolarization spread and measured both in experiments and simulations the propagation speed. The model served also as a support for the experimental findings in which the intracellular Ca^2+^ sources were not involved in the generation of ultrafast Ca^2+^ wave. In our model, VOCCs were necessary for Ca^2+^ entry after depolarization. This Ca^2+^ influx event progressively propagated along the SMCs network with an intensity depending on the amplitude of the membrane depolarization. Importantly, the model predicted that a membrane depolarization was still present even when the VOCCs were inactive. It also allowed us to estimate valid ranges of critical parameters that are challenging to measure experimentally, such as electrical coupling coefficient and whole-cell conductance for VOCCs. Finally, the model reproduced, validated and allowed a better understanding of recently reported experimental data. In the context of the vascular system, analysis and modeling of SMCs mechanisms should ultimately provide new insights into the dynamic coordination of vasculature and thereby contribute to the regulation of tissue perfusion.

## Methods

### Experimental methods

**Figure 6:**
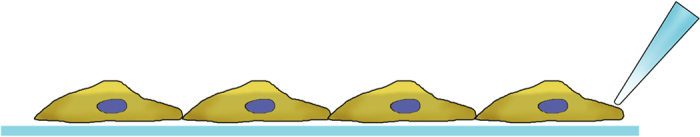
Local mechanical stimulus was performed with a micropipette (1 *μ*m tip diameter, Eppendorf Femtotips I).

**Figure 7:**
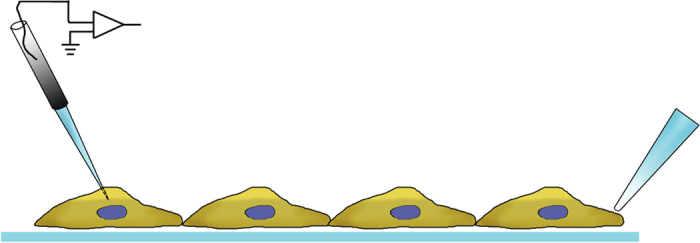
Intracellular recordings of vascular SMCs membrane potential were performed with microelectrodes made from borosilicate glass tubing containing an inner glass filament (World Precision Instruments, Sarasota, FL, USA) and pulled on a horizontal puller P-2000 (Sutter Instruments, Novato, CA, USA).

The cell culture of primary SMCs (pSMCs) and rat aortic SM cell line (A7r5), pattern microfabrication, mechanical stimulation protocols (Fig. 6), Ca^2+^ fluoroscence and membrane depolarization measurements with microelectrode technics (Fig. 7), were previously detailed in ref. 18.

#### Chemical and drugs

We used the drug 2-Aminoethoxy-diphenylborate (2-APB) (EMD Millipore, Billerica, MA, USA) that blocks IP_3_ receptors. 2-APB was first dissolved at 100 mM in DMSO, and then diluted to 30 *μ*M in buffer solution. Cells were allowed to preincubate in 2-APB for 10 minutes.

Ryanodine (20 *μ*M), PA (50 *μ*M), Nifedipine (10 *μ*M).

#### Velocity measurements of membrane depolarization spreading

One of the important features in the use of the microelectrodes technique in combination with a precise patterned growth of cells and a reproducible way of mechanical stimulation is the possibility to measure the conduction velocity of the membrane depolarization. The local mechanical stimulation was performed using a high speed programmable micromanipulator for cell injection equipped with a micropipette. The electrical recordings were collected through a microelectrode; the conditioned signal was recorded with an analog-to-digital acquisition card (NI-USB 6009, National Instruments Corporation). A custom Matlab program (The MathWorks, Inc.) controlled simultaneously the micromanipulator via the serial interface and the acquisition card, handling the synchronization of the mechanical stimulation and the recording of the running depolarization. Thus, performing twice the mechanical stimulation in the same cell with the same conditions and registering the changes in the membrane potential for two different cells within the cell network, it was possible to estimate the spreading velocity of the membrane depolarization. The spreading velocity is calculated by dividing the difference in the onset times of membrane depolarization for the two different membrane potential registrations (two different cells in the same network) by the separation distance between them. The crucial part that allowed the conduction velocity calculation was the development of a program that allowed us to have a recording system that controls both the moment of stimulation and the registration of electrical events.

### Analysis of experimental data

The experimental data presented here are expressed as means ± SEM (n). Statistical significance was tested using Student’s t-test on paired data; P < 0.05 was considered significant; n represents the number of experiments.

### Numerical methods

The equations describing the cytosol [Ca^2+^] and the membrane potential dynamics in SMCs were taken from^11^. This model takes into account the most relevant cellular mechanisms involved during the generation of Ca^2+^ increases^33^. The model equations were solved using a fourth-order Runge-Kutta method and integrated on a one-dimensional line of SMCs comprising 100 cells. Within each SMC, the Ca^2+^ and the membrane potential dynamics are described by Eqs 1, 2, 3, 4, 5 and each cell was connected with its nearest left and right neighbors via electrical coupling (neglecting intracellular propagation of membrane potential). Local mechanical^34^ or KCl stimulation^4^ induced membrane depolarization. We mimicked the single cell stimulation process in the model by changing the reversal potential of K^+^ and Cl^−^ (v_*K*_ and v_*Cl*_) in Eqs 14 and 15 of the first cell. Extracellular KCl changed the Nernst potentials for K^+^ and Cl^−^, depolarizing the cell membrane of the stimulated SMC^35^. We translated the unit mm.s^−1^ to cells.s^−1^ assuming a typical length for a single SMC of 100–150 *μ*m, estimated from the cells spatial configuration used in ref. 18. In the simulations, ~16 cells correspond approximatively to the number of cells in the experiments.

### Model of coupled Smooth Muscle Cells

The Ca^2+^ dynamics in each SMC (*i*), is described by five variables: the Ca^2+^ concentration in the cytosol (*c*_*i*_); the Ca^2+^ concentration in the sarcoplasmic reticulum (*s*_*i*_); the cell membrane potential (*v*_*i*_); the open-state probability of Ca^2+^ activated potassium channels (*w*_*i*_) and the IP_3_ concentration (*I*_*i*_). Cells are connected to their nearest neighbors via gap-junctional electrical coupling.

The mathematical approach for modeling the Ca^2+^ dynamics and electrophysiology in a SMC is given by the following equations:


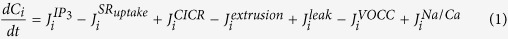



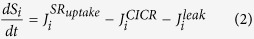



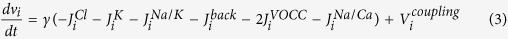



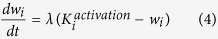



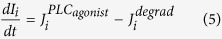


The equations (1, 2, 3, 4, 5) are detailed in refs 11 and 33.

*J*^*VOCC*^ models the Ca^2+^ influx through VOCCs:


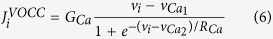



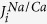
 is the Na^+^/Ca^2+^ exchange:


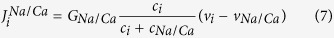



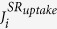
 represents the SR uptake:


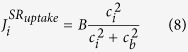



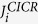
 corresponds the Ca^2+^ induced Ca^2+^ release (CICR):


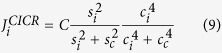



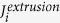
 models the Ca^2+^ extrusion from the SMCs by the Ca^2+^ -ATPase pumps:


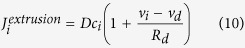



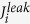
 is the leak from SR:


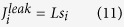



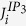
 represents the Ca^2+^ release from IP_3_-sensitive stores:


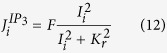



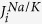
 models the Na^+^-K^+^-ATPase:


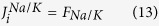



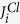
 corresponds to the chloride channels and it takes into account that they are Ca^2+^ -activated;


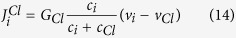



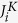
 is the K^+^ efflux:


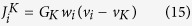



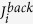
 represents the background currents:


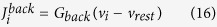



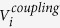
 models the electrical coupling:


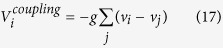



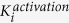
 represents the Ca^2+^ and the voltage activation of the K^+^ channels:


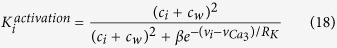



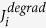
 models the IP_3_ degradation:


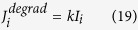


### Model parameters

Model parameters were taken from^11^ except the whole-cell conductance for background current G_back_ and SR uptake constant *B* values to avoid spontaneous oscillations and ensure the stability of the system during the stimulation.

For the control case we fixed the whole-cell conductance for VOCCs G_Ca_ = 0,036195 *μ*.M.mV^−1^.s^−1^ and the value of the electrical coupling coefficient g = 1000 s^−1^. The latter corresponds to the gap-junctional conductance measured experimentally^36^,^37^,^38^,^39^. The gap junctional electrical coupling coefficient *g* is related to the gap junctional conductance G by g = G/C_m_, where C_m_ is the cell membrane capacitance.

To further investigate the effect these two crucial parameters on the wave, we used *g* from 10 to 5000 s^−1^ and G_Ca_ from 0,026 to 0,0525 *μ*M.mV^−1^.s^−1^.[Table t1]

## Additional Information

**How to cite this article**: Quijano, J. C. *et al*. Intercellular ultrafast Ca^2+^ wave in vascular smooth muscle cells: numerical and experimental study. *Sci. Rep*. **6**, 31271; doi: 10.1038/srep31271 (2016).

## Supplementary Material

Supplementary Information

## Figures and Tables

**Table 1 t1:** List of parameters

Parameter	Description	Value
F	Maximal rate of activation-dependent Ca^2+^ influx^11^	3,45 *μ*M.s^−1^
K_*r*_	Half-saturation constant for agonist-dependent Ca^2+^ entry^11^	1,0 *μ*M
G_*Ca*_	Whole-cell conductance for VOCCs^11^	0,036195 *μ*M.mV^−1^.s^−^1
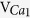	Reversal potential for VOCCs	100,0 mV
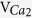	Half-point of the VOCC activation sigmoidal^11^	−24,0 mV
R_*Ca*_	Maximum slope of the VOCC activation sigmoidal^11^	8,5 mV
G_*Na*/*Ca*_	Whole-cell conductance for the Na^+^/Ca^2+^ exchanger^11^	0,006 *μ*M.mV^−1^.s^−1^
c_*Na*/*Ca*_	Half-point for activation of Na^+^/Ca^2+^ exchanger by Ca^2+ ^11	0,5 *μ*M
v_*Na*/*Ca*_	Reversal potential for the Na^+^/Ca^2+^ exchanger^11^	−30,0 mV
B	SR uptake rate constant	2,025 *μ*M.s^−1^
c_*b*_	Half-point of the SR ATPase activation sigmoidal^11^	1,0 *μ*M
C	CICR rate constant^11^	1545,0 *μ*M.*s*^−1^
s_*c*_	Half-point of the CICR Ca^2+^ efflux sigmoidal^11^	2,0 *μ*M
c_*c*_	Half-point of the CICR activation sigmoidal^11^	0,9 *μ*M
D	Rate constant for Ca^2+^ extrusion by the ATPase pump^11^	3,6 s^−1^
v_*d*_	Intercept of voltage dependence of extrusion ATPase	−100,0 mV
R_*d*_	Slope of voltage dependence of extrusion ATPase^11^	250,0 mV
L	Leak from SR rate constant^11^	0,375 s^−1^
*γ*	Scaling factor relating net movement of ion fluxes^11^	492,5 mV.*μ*M^−1^
	membrane potential (inversely related to cell capacitance)	
F_*Na*/*K*_	Net whole-cell flux via the Na^+^-K^+^-ATPase^11^	0,03 *μ*M.s^−1^
G_*Cl*_	Whole-cell conductance for Cl^−^ current^11^	0,6 *μ*M.mV^−1^.s^−1^
c_*Cl*_	Ca^2+^ sensitivity for Cl^−^ channels^11^	0,7 *μ*M
v_*Cl*_	Reversal potential for Cl^−^ channels^11^	−25,0 mV
G_*K*_	Whole-cell conductance for K^+^ efflux^11^	0,045 *μ*M.mV^−1^.s^−1^
v_*K*_	Reversal potential for K^+ ^11	−94 mV
*λ*	Rate constant for net KCa channel opening^11^	675
c_*w*_	Translation factor for Ca^2+^ dependence of K_*Ca*_^11^	0,0 *μ*M
	channel activation sigmoidal^11^	
*β*	Translation factor for the membrane potential dependence^11^	0,001 *μ*M^2^
	of K_*Ca*_ channel activation sigmoidal^11^	
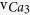	Half-point for the KCa channel activation sigmoidal^11^	−27,0 mV
R_*K*_	Maximum slope of the KCa activation sigmoidal^11^	12,0 mV
G_*back*_	Whole-cell conductance for background currents^40^,^41^	0,0045 *μ*M.mV^−1^.s^−1^
v_*rest*_	Equilibrium potential^11^	−55,0 mV
k	Rate constant of IP_3_ degradation^11^	0,1 s^−1^
g	Electrical coupling coefficient^11^	1000 s^−1^
