# Supplementary material for: Intercellular ultrafast Ca2+ wave in vascular smooth muscle cells: numerical and experimental study
Source: Sci Rep. 2016 Aug 10;6:31271. doi: 10.1038/srep31271 (PMC4978975; doi:10.1038/srep31271)
Supplement: Supplementary Information [file srep31271-s1.pdf]

Intercellular ultrafast Ca^{2+} wave in vascular smooth muscle cells: numerical and experimental study

J. C. Quijano,[1,2] F. Raynaud,[1,3] D. Nguyen,[1] N. Piacentini,[1] and J. J. Meister[1]

[1]*Laboratory of Cell Biophysics, Ecole Polytechnique Fédérale de Lausanne, Lausanne, Switzerland*
[2]*Facultad de Ciencias Básicas, Politécnico Colombiano JIC, Medellín, Colombia*
[3]*Computational Biology Cancer Genomics, Department of Computational Biology, University of Lausanne, Lausanne, Switzerland*

Supplementary Information

Supplementary Fig. S1 summarizes experimental results obtained on a precise linear arrangement of cultured SMCs in [1]. Experimental phase contrast images encompass a line of a length of $\sim 1,5$ mm (Supplementary Fig. S1A). Local mechanical stimulation was performed in a single SMC and induced a fast initial increase in [Ca^{2+}] along the network that can be interpreted as an ultrafast Ca^{2+} wave. Ca^{2+} responses were obtained in the stimulated cell and in other three regions of interest (ROIs) along the line of cells (Supplementary Fig. S1B). The experimental setup was designed to measure delay in time between ROIs. This experimental procedure allowed reliable measurements of intercellular speed by taking ROIs separated enough. Therefore this approach is not suitable for the calculation of intracellular Ca^{2+} wave speed. Inhibitors of gap junctions and of voltage-operated Ca^{2+} channels (VOCCs) abolished the wave (Fig. 1C,D). Incubation of the cells preparation with the ryanodine receptors (RyRs) inhibitor ryanodine did not affect the wave (Supplementary Fig. S1E). The range of the ultrafast Ca^{2+} wave was $\sim 1,5$ mm and its velocity 15 mm.s^{-1} [1].

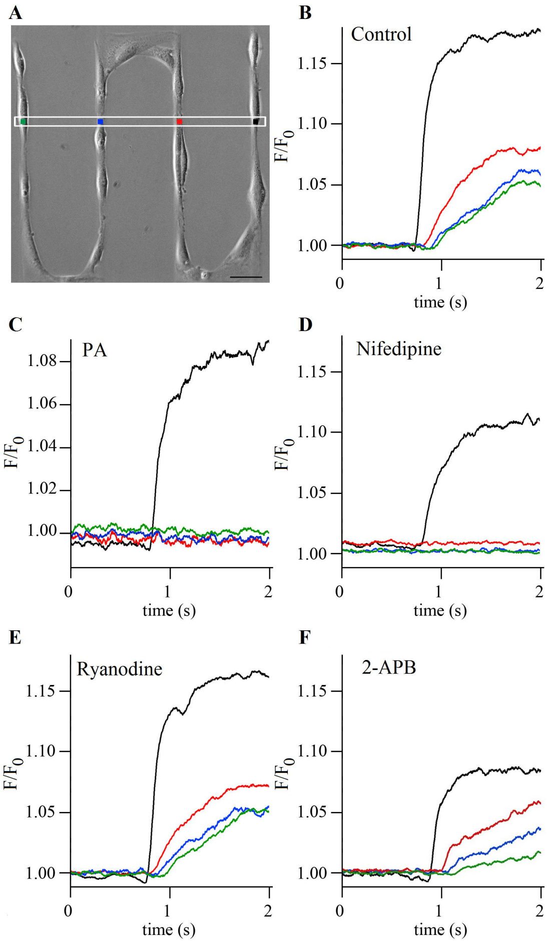

Supplementary Figure 1: Mechanical stimulation induced ultrafast Ca^{2+} wave in cultured SMCs. A) Phase contrast images of the cultured SMCs in a line pattern configuration. To analyze the intercellular ultrafast Ca^{2+} wave, four ROIs were taken along the linear arrangement of SMCs. The black ROI corresponds to the stimulated cell, the 2nd, 3rd and 4th ROIs are represented in red,blue and green. The fluorescence ratio (F/F_0) were measured in each ROI (squares of nine pixels), F_0 is the fluorescence intensity before stimulation. Scale bar: 50μm. B) Time evolution of the Ca^{2+} fluorescence intensity in the colored ROIs selected in A. Experiments in the presence of gap junction inhibitor (palmitoleic acid, 50μM) C) and VOCCs inhibitor (nifedipine, 10μM) D) showed no fluorescence ratio increase, except in the stimulated cell. When the cell preparation was incubated with the RyRs inhibitor ryanodine, 20μME) or IP_3Rs inhibitor 2-APB F), there was no significant difference with the control case. Modified from [1]

[1] J. C. Quijano, B. Vianay, J. L. Bény, and J. J. Meister, Cell Calcium **54**, 436 (2013), ISSN 01434160.
